# The meaning of biological information

**DOI:** 10.1098/rsta.2015.0065

**Published:** 2016-03-13

**Authors:** Eugene V. Koonin

**Affiliations:** National Center for Biotechnology Information, National Library of Medicine, National Institutes of Health, Bethesda, MD, USA

**Keywords:** information, meaning, evolution, selfish elements

## Abstract

Biological information encoded in genomes is fundamentally different from and effectively orthogonal to Shannon entropy. The biologically relevant concept of information has to do with ‘meaning’, i.e. encoding various biological functions with various degree of evolutionary conservation. Apart from direct experimentation, the meaning, or biological information content, can be extracted and quantified from alignments of homologous nucleotide or amino acid sequences but generally not from a single sequence, using appropriately modified information theoretical formulae. For short, information encoded in genomes is defined vertically but not horizontally. Informally but substantially, biological information density seems to be equivalent to ‘meaning’ of genomic sequences that spans the entire range from sharply defined, universal meaning to effective meaninglessness. Large fractions of genomes, up to 90% in some plants, belong within the domain of fuzzy meaning. The sequences with fuzzy meaning can be recruited for various functions, with the meaning subsequently fixed, and also could perform generic functional roles that do not require sequence conservation. Biological meaning is continuously transferred between the genomes of selfish elements and hosts in the process of their coevolution. Thus, in order to adequately describe genome function and evolution, the concepts of information theory have to be adapted to incorporate the notion of meaning that is central to biology.

## 1. Entropy, information, meaning and genome evolution

One of the most common, textbook concepts in biology is that the genome encodes information on the organism—or synonymously, the genotype encodes information about the phenotype. Genomes are ultimately strings of symbols, and this digital organization is naturally interpretable within the framework of standard information theory [1,2]. The classical Shannon formula for the mean entropy (often interpreted as information content) per position of a nucleotide (or amino acid) sequence of length *L* be written as
1.1
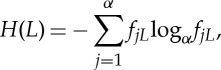
where *f*_*j*_ is the frequency of the base *j* ( *j*=A,T,G,C) in the given sequence, and *α* is the size of the alphabet (four in the case of nucleotide sequences and 20 for amino acid sequences). Applied this way, entropy only tells us how far the count of each base in the given sequence deviates from the random expectation *L*/*α* which does not convey any meaningful message on the genome in question let alone about the phenotype of the organism it is supposed to encode. Clearly, the message encoded in the genome is of a different nature. Within the classical information theory, the quantity we are interested in is not entropy but rather information (more precisely, information gain) that is obtained about a sequence *L* as a result of some procedure that we will call measurement:
1.2
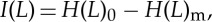
where *H*(*L*)_0_ is the entropy of the sequence before the measurement and *H*(*L*)_m_ is the entropy of the same sequence after the measurement. An insightful discussion of the crucial distinction between information and entropy is presented by Adami in this theme issue of the *Philosophical Transactions*[3].

In qualitative terms, biological information is perhaps best described as the ‘meaning’ of a sequence. A nucleotide sequence assumes meaning only when it is either transcribed into a RNA molecule that directly carries out a biological function, or transcribed into a mRNA that is then translated into a functional protein, or else the DNA itself interacts with proteins or RNA molecules resulting in a functional (often, regulatory) effect.

What kind of measurements can yield biological information and allow one to quantify the meaning of genomic sequences? Obviously, one of the means to this end is direct experimentation. However, exhaustive characterization of the biological roles of each nucleotide in the genome is unrealistic even for the smallest model genomes such as those of viruses, let alone the expansive genomes of complex organisms such as animals and plants. Moreover, quantitative comparison of the ‘meaningfulness’ of different sites in the genome requires a whole other level of experimentation whereby the phenotypic (fitness) effects of changes in each site are measured in competition experiments. This type of experiment is central to experimental evolution research [4,5] but the complexity of bringing it to the genome scale far exceeds any imaginable laboratory capabilities.

Hence the alternative approach to information measurement involves extracting meaning from sequences themselves. A single genomic sequence is largely meaningless. The meaning of certain short nucleotide signals has been known for many years from multiple, definitive experiments. The most prominent signals of this type are the translation start and stop codons that mark protein-coding genes. A simple estimate shows that the presence of a long open reading frame between a start and a stop signal is highly unlikely, and therefore, at least for intron-less genomes, protein-coding regions can be predicted reliably [6]. This does tell us something important about the meaning of the genome sequence, by delineating regions that most likely are used to produce proteins.

However, the only general way to extract meaning from sequences involves comparative analysis of homologues. The premises are extremely simple, yet powerful. The great majority of the meaningful sites in nucleotide sequences, i.e. those sites that contribute to biological function, are subject to purifying selection, hence evolutionary conservation of meaningful sites. The stronger the selection, the more meaningful (‘important’) a site is. These simple considerations allow one to naturally quantify meaningful information contained in sequences [7–10].

For an alignment of orthologous sequences, the Shannon entropy formula (1.1) can be re-written as follows:
1.3
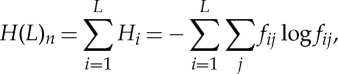
where *H*(*L*)_*n*_ is the total entropy of the alignment of *n* sequences of length *L*; *H*_*i*_ is the per site entropy; and *f*_*ij*_ are the frequencies of each of the four nucleotides ( *j*=A,T,G,C) or each of the 20 amino acids in site *i*. Clearly, for a fully conserved site *H*(*i*)=0, whereas for a completely random site *H*(*i*)=1; accordingly, the values of *H*(*L*)_*n*_ are between 0 and *L*. Note that equation (1.3) is equivalent to equation (1.1) except that instead of applying the Shannon formula ‘horizontally’, i.e. to a single sequence, we now apply it ‘vertically’, i.e. to an alignment of homologous sequences. This definition of entropy is consistent both with Boltzmann's famous statistical definition of entropy and with Shannon entropy (information content) and thus can be legitimately denoted ‘evolutionary entropy’ of a set of aligned sequences. In addition to being physically valid, evolutionary entropy seems to make perfect biological sense: low-entropy sites are most strongly conserved, and by inference, most functionally important (meaningful).

Then, using formula (1.3), ‘biological (evolutionary) information’ of a genome can be defined as
1.4
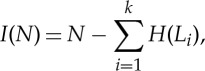
and ‘biological (evolutionary) information density’ can be calculated as
1.5
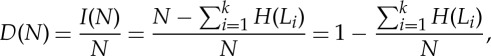
where *N* is the total length (number of sites) in a genome; *L*_*i*_ is the length of a genomic segment that is subject to measurable selection (such as a protein-coding or RNA-coding gene); *k* is the number of such alignable segments in the genome; and *H*(*L*_*i*_) is the evolutionary entropy for the segment *L* calculated using formula (1.2). Previously, the quantity defined by equation (1.4) has been denoted ‘biological complexity’ but at least for the purpose of the present discussion, ‘biological (evolutionary) information’ seems to be a more straightforward definition. The values of *I*(*N*) are between 0 and *N*, and equation (1.4) is equivalent to equation (1.2), i.e. biological information is the information gain that can be extracted from an alignment of homologous sequences through the constraint on change in ‘meaningful’ positions. Indeed, biological information density is directly related to meaning: sites and sequences with the highest values of *D*(*N*) are the most meaningful ones. Thus, Dobzhansky's famous dictum ‘Nothing in biology makes sense except in light of evolution’ [11] takes a literal, even technical interpretation: biological meaning (sense) effectively cannot be gleaned by any means other than direct evolutionary analysis.

To conclude this conceptual discussion, it seems pertinent to ask: what is biological information about? It has been persuasively argued that the genome stores information about the environment, allowing the organism to predict and exploit environmental changes [10,12]. Although environmental interactions certainly are an important part of the genomic information content, it seems prudent to indicate that another key part is about the (nearly) universal aspects of cellular and organismal design. A notable evidence of the universality of cellular design comes from the consistent observations on the universal scaling of different functional categories of genes with the total gene count in all cellular life forms [13,14]. The genes encoding universal components of cells, such as RNA and proteins that constitute the translation system, are endowed with by far the most pronounced meaning (i.e. evolutionary conservation) than genes that are involved in environmental interactions [15,16].

## 2. Informational and entropic genomes and evolution of organismal complexity

The exact values of *H* are difficult to calculate for complete genomes because the distribution of evolutionary constraints is never known precisely [16]. Furthermore, there is always arbitrariness in the choice of orthologues to be included in the alignment for the calculation, and most important, the sequences of orthologous genes are actually not independent but rather are connected by an evolutionary tree. Thus, to produce accurate estimates of biological information density, an appropriate weighting scheme taking into account the evolutionary tree topology and branch lengths is required. However, these details are not essential if one is interested only in ballpark estimates. The fraction of sites under selection across the genome has been estimated with reasonable precision for some model organisms such as humans or *Drosophila* [16–18]. For others, particularly prokaryotes and unicellular eukaryotes, the fraction of coding nucleotides plus the estimated fraction of regulatory sites can be taken as a reasonable approximation; for sites under selection, *H*_*i*_=0.5 can be taken to approximate the mean entropy value.

**Figure 1.**
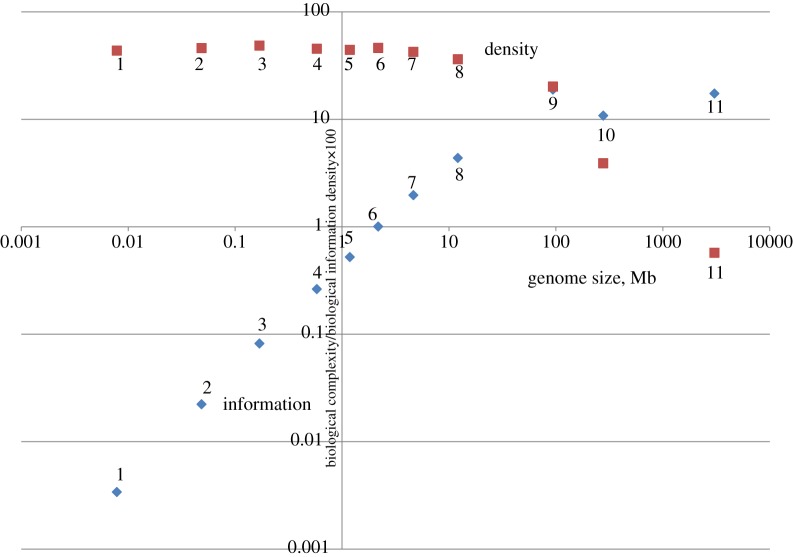
Biological information and information density depending on genome size: viruses, prokaryotes and eukaryotes. The biological information and density values were calculated using equations (1.4) and (1.5), respectively, and the data on genomes were from Genbank. The plot is on a double logarithmic scale. 1, encephalomyocarditis virus (RNA virus); 2, lambda phage; 3, T4 phage; 4, *Mycoplasma genitalium* (parasitic bacterium); 5, acanthamoeba polyphaga mimivirus (giant virus); 6, *Archaeoglobus fulgidus* (free-living archaeon); 7, *Escherichia coli* (free-living bacterium); 8, *Saccharomyces cerevisiae*; 9, *Arabidopsis thaliana*; 10, *Drosophila melanogaster*; 11, *Homo sapiens*. (Online version in colour.)

Comparison of the estimates of *H*(*N*), *I*(*N*) and *D*(*N*) for genomes of different life forms reveals a paradox. The total biological information *I*(*N*) (arguably, the measure of biological complexity) monotonically increases with the genome size, in particular, in multicellular eukaryotes compared to prokaryotes, but the entropy *H*(*N*) increases dramatically faster, and as the result, the evolutionary information density *D*(*N*) sharply drops (figure 1). Thus, the genomes of organisms that are usually perceived as the most complex, such as animals and plants, indeed have the highest total information content but also are ‘entropic’ genomes with a low biological information density. By contrast, organisms that we traditionally think of as primitive, such as bacteria, have ‘informational’ genomes with high information density [9]. To rephrase the same statement more provocatively, the genomes of unicellular organisms and viruses appear to be incomparably ‘better designed’ than the genomes of plants or particularly animals. Certainly, this conundrum is already apparent in a simple comparison of the genome architectures of multicellular and unicellular organisms, with the former being dominated by non-coding sequences (introns and intergenic regions), whereas the latter are ‘wall to wall’ genomes that are almost completely comprised of genes [19]. Nevertheless, the formal approach to biological information outlined above allows one to emphasize and quantify the differences between the informational landscapes of different life forms.

The primary cause behind the low information density of the genomes of the complex life forms seems to follow directly from straightforward population genetic theory [20–22]. In populations with a small effective size that are characteristic of complex multicellular organisms, the weak purifying selection and the high intensity of genetic drift preclude efficient purging of meaningless sequences and conversely allow proliferation of such sequences, in particular, various mobile elements. Evolutionary and functional plasticity is the other side of the same coin [16]. This plasticity is manifested in the numerous demonstrated cases of recruitment of mobile element sequences and other originally ‘meaningless’ sequences for biological functions. What matters for the evolution of phenotypic (organismal) complexity appears to be the total biological information content of the genome rather than information density (‘design’). I discuss these aspects of biological information in the following sections.

## 3. Junk DNA or sequences with fuzzy meaning?

**Figure 2.**
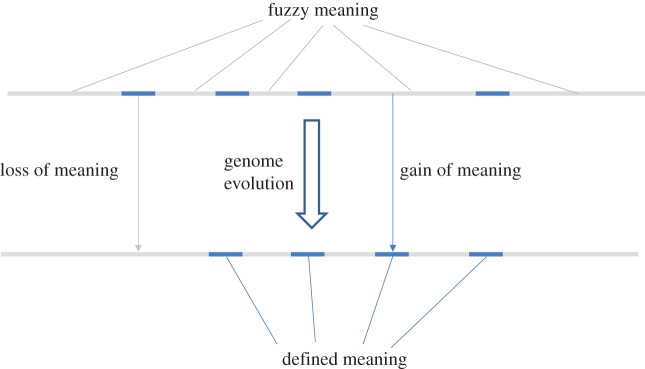
The fuzzy meaning concept and gain and loss of meaning. The cartoon schematically shows a fragment of a genome of a complex multicellular organism (animal or plant) that consists mostly of sequences with fuzzy meaning, interspersed with ‘islands’ of defined meaning such as genes (exons) encoding structural RNAs and proteins as well as evolutionarily conserved regulatory elements. (Online version in colour.)

The now well-established phenomenon of pervasive transcription [23–25], that has triggered the (in)famous debate around the results of the ENCODE project [26–30], when pitted against the formal considerations outlined above, suggests a radical line of thinking on the nature of biological information and meaning. The indisputable findings that (nearly) all sequences in complex genomes, such as human, are transcribed at some level (at least in some cell types and at some life stages) most likely fit the same population genetic perspective [20–22]. Conceivably, transcription is pervasive because selection against spurious promoters and enhancers is not sustainable in small populations subject to drift. However, in the context of the above formalization of biological information, would it be appropriate to view most of the sequences in complex genomes, with (extremely) low biological information density, as being endowed with ‘fuzzy meaning’ (figure 2)? Operationally, sequences with fuzzy meaning can be defined as those that cannot be aligned between genomes that have diverged beyond the threshold of sequence conservation that is due to common ancestry alone, e.g. after the synonymous sites in protein-coding genes have reached saturation. This is a conservative definition because it assumes neutrality of the synonymous sites that in actuality are subject to selection albeit substantially weaker than that affecting non-synonymous sites [31–33].

The exact sequences of genomic regions with fuzzy meaning are (almost) inconsequential but their expression has meaning that can be rationalized at least at two levels. First, the sequences with fuzzy meaning form the material basis of plasticity from which functional molecules, primarily but not exclusively, regulators of various processes, are continuously recruited to assume better defined meaning, a process that can be denoted ‘gain of meaning’. Second, although numerous sequences might not encompass any specific meaning whatsoever, their transcription itself could be meaningful, in particular, for maintaining particular chromatin states that in turn regulate transcription of regions with specific meaning (genes) [24]. The information flow between the domains of defined meaning and fuzzy meaning certainly is a two-way street: loss of meaning continuously occurs, e.g. in the process of pseudogenization.

The boundary between the genomic sequences with well-defined meaning and those with fuzzy meaning is not necessarily sharp. The long non-coding (lnc) RNAs that recently have been identified in abundance in mammals [34–36] appear to bridge the islands of highly meaningful protein-coding regions (and those that encode structural RNAs) with the sea of fuzzy meaning sequences (figure 2). In the expansive pool of lncRNAs, there are many that are represented by orthologues even in distant organisms, such as primates and rodents, although sequence conservation (biological information density) is low [35,37–40]. However, numerous lncRNAs, even within well-defined, relatively highly expressed sets [39], are lineage-specific and hence belong in the fuzzy meaning domain.

The concept of fuzzy meaning seems to reconcile two fundamental, undeniable but apparently contradictory lines of evidence: (i) in complex, large genomes, the substantial majority of the sequences is subjected to extremely weak or effectively no purifying selection and (ii) most of these apparently meaningless sequences are at least occasionally transcribed, i.e. have a distinct phenotypic manifestation. Rather than dismissing most of the genome as junk DNA [41–44], the fuzzy meaning concept seems to offer a more adequate description of this vast pool of sequences.

The relevance of fuzzy meaning for evolution, and in particular evolutionary innovations, does not appear to be limited to non-coding DNA or to large, complex genomes. It has been observed that most of the novel eukaryotic proteins adopt *α*-helical folds and seem to have evolved from generic, repetitive coiled coil proteins [45]. Even more strikingly, evidence has been presented that in various eukaryote organisms, numerous short genes evolve from non-coding sequences through a stage of ‘pre-proteins’ [46–52]. This route of evolution appears to be a clear manifestation of fuzzy meaning. Upon acquiring a specific function, the evolution of these proteins substantially slowed down: their meaning was sharply defined as they left the fuzzy domain (figure 2).

## 4. The agency of biological meaning: parasite–host interaction, arms races and exaptation

Much like in human affairs and unlike in standard information theory, the meaning of genomic sequences can only be meaningfully defined if the beneficiary of the message is identified (‘meaning for whom?’). The sequences of the innumerable selfish, mobile genetic elements that are integrated into the genomes of cellular lifeforms [53–57]—and represent the majority of the genome sequences in many animals and plants [58,59]—are generally meaningless for the host organisms. For most of these elements, orthologous relationships cannot be established between any distant host species, and hence biological information density cannot be estimated from the host genome comparison. Yet, ‘from the selfish element's point of view’, i.e. when biological information density is estimated from an alignment of homologous sequences of elements in the same family, these sequences are densely packed with meaning.

Continuous transfer of meaning between selfish elements and hosts is a major evolutionary trend that comes in several guises. First, genes from selfish elements are often recruited by host organisms such that the specific activity of the encoded protein is modified and appropriated for host functions. Examples include the essential eukaryotic enzyme telomerase that is required for linear chromosome replication that was recruited from a bacterial retroelement (group II self-splicing intron) for its reverse transcriptase activity [60,61]; hedgehogs, key regulators of animal development, that have been derived from an intein and employ the autoprotease activity of the latter [62–64]; and syncytins, placental receptors derived from retrovirus genes [65,66]. A remarkable, common phenomenon involves what can be described as a change of biological meaning to its opposite. Under this trend, ‘offensive weapons’ of selfish elements are captured by the hosts and turned into means of defence [67]. Striking examples include the parallel recruitment of integrases from unrelated selfish elements for adaptive immunity systems in prokaryotes (CRISPR-Cas) and in animals [68] as well as the system of DNA elimination in the ciliate macronucleus [69]. Conversely, selfish elements, particularly viruses with comparatively large genomes, consistently capture host genes involved in defence and adopt them for counter-defence, e.g. as dominant-negative inhibitors [70–72]. Thus, sequences that have been meaningful for selfish elements become meaningful for the host and vice versa.

Another common trend in the coevolution of selfish elements and hosts is the erosion of meaning that accompanies integration of genomes. This phenomenon includes inactivation and deterioration of all kinds of mobile elements that occurs on a limited scale in bacteria and archaea but is a massive contribution to the genomes of animals and plants. Including splicesosomal introns which appear to be descendants of bacterial retrotransposons (group II self-splicing introns) [61], the majority of the DNA in animal and plant genomes is derived from mobile elements. Thus, these elements are the principal source of fuzzy meaning discussed in the preceding section.

## 5. Conclusion

**Figure 3.**
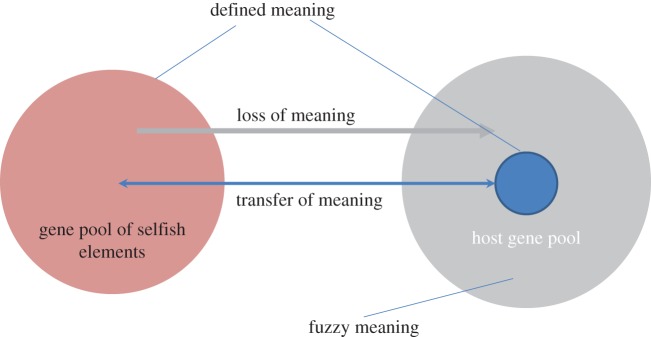
Flow of meaning between selfish elements and hosts. (Online version in colour.)

The biologically relevant information is more akin to meaning than to entropy. This type of information can be quantified by applying theoretical informational concepts to aligned sequences of orthologous genes or proteins: biological information density (meaning) is defined vertically, i.e. across an alignment of homologous sequences, rather than horizontally, i.e. along a single genome. Sites with the lowest entropy have the highest biological information density or in other words, are the most meaningful ones. The meaningfulness of genomic sequences spans the entire range from sharply defined, universal meaning to effective meaninglessness. Sequences with low biological information density can be assigned to the domain of fuzzy meaning which encompasses most of the genomic sequence in animals and plants. The sequences with fuzzy meaning serve as a pool for recruitment for diverse functions and could also be involved in generic functional roles that require little if any sequence conservation. The concept of fuzzy meaning seems to capture better the status of non-conserved genomic sequences than the more rigid notion of junk DNA. The evolution of life involves the perennial arms race between parasites and hosts that involves continuous transformation of the agency of biological meaning. Sequences that are meaningful for selfish elements are appropriated by the hosts to assume meaning, particularly as means of defence, and vice versa, host genes involved in antivirus defence and other processes are recruited by selfish elements, with their meaning changed in the process. An even more common phenomenon is the erosion of meaning of selfish element genes upon integration with the host genomes. These sequences replenish the fuzzy meaning domain. In summary, meaningful analysis of genomes from an informational theoretical standpoint requires re-interpretation of the very notion of information as a concept of meaning that is specific to biology (figure 3).
